# Pharmacovigilance evaluation of the relationship between impaired glucose metabolism and BCR‐ABL inhibitor use by using an adverse drug event reporting database

**DOI:** 10.1002/cam4.1920

**Published:** 2018-12-18

**Authors:** Naoto Okada, Takahiro Niimura, Yoshito Zamami, Hirofumi Hamano, Shunsuke Ishida, Mitsuhiro Goda, Kenshi Takechi, Masayuki Chuma, Masaki Imanishi, Keisuke Ishizawa

**Affiliations:** ^1^ Department of Pharmacy Tokushima University Hospital Tokushima Japan; ^2^ Department of Clinical Pharmacology and Therapeutics Tokushima University Graduate School of Biomedical Sciences Tokushima Japan; ^3^ Clinical Trial Center for Developmental Therapeutics Tokushima University Hospital Tokushima Japan

**Keywords:** BCR‐ABL inhibitors, FAERS, impaired glucose metabolism, JADER, spontaneous reporting system

## Abstract

Breakpoint cluster region‐Abelson murine leukemia (BCR‐ABL) inhibitors markedly improve the prognosis of chronic myeloid leukemia. However, high treatment adherence is necessary for successful treatment with BCR‐ABL inhibitors. Therefore, an adequate understanding of the adverse event profiles of BCR‐ABL inhibitors is essential. Although many adverse events are observed in trials, an accurate identification of adverse events based only on clinical trial results is difficult because of strict entry criteria or limited follow‐up durations. In particular, BCR‐ABL inhibitor‐induced impaired glucose metabolism remains controversial. Pharmacovigilance evaluations using spontaneous reporting systems are useful for analyzing drug‐related adverse events in clinical settings. Therefore, we conducted signal detection analyses for BCR‐ABL inhibitor‐induced impaired glucose metabolism by using the FDA Adverse Event Reporting System (FAERS) and Japanese Adverse Drug Event Report (JADER) database. Signals for an increased reporting rate of impaired glucose metabolism were detected only for nilotinib use, whereas these signals were not detected for other BCR‐ABL inhibitors. Subgroup analyses showed a clearly increased nilotinib‐associated reporting rate of impaired glucose metabolism in male and younger patients. Although FAERS‐ and JADER‐based signal detection analyses cannot determine causality perfectly, our study suggests the effects on glucose metabolism are different between BCR‐ABL inhibitors and provides useful information for the selection of appropriate BCR‐ABL inhibitors.

## 1 INTRODUCTION

Chronic myeloid leukemia (CML) is a clonal stem cell neoplasm characterized by the presence of the Philadelphia (Ph) chromosome.^1^ The Ph chromosome produces the breakpoint cluster region‐Abelson murine leukemia (BCR‐ABL) fusion protein, which dysregulates tyrosine kinase activity and induces uncontrolled proliferation of the granulocyte lineage. Imatinib, nilotinib, dasatinib, bosutinib, and ponatinib are tyrosine kinase inhibitors that inhibit the activity of the BCR‐ABL fusion protein. These BCR‐ABL inhibitors have been reported to improve markedly the prognosis of CML.^2^, ^3^, ^4^, ^5^, ^6^ However, maintaining high therapeutic adherence is necessary to obtain the maximum therapeutic effect with BCR‐ABL inhibitors.^7^ For instance, an adequate blood concentration of imatinib is necessary to obtain the maximum therapeutic effect.^8^ However, adverse events induced by BCR‐ABL inhibitors decrease therapeutic adherence. Therefore, a good understanding of the adverse event profile of BCR‐ABL inhibitors is essential for successful CML treatment.

Adverse events related to a class effect or a drug effect of BCR‐ABL inhibitors have been observed in clinical trials. Cardiovascular toxicity was reported as a common adverse event induced by all BCR‐ABL inhibitors,^9^ while impaired glucose metabolism was reported in cases of nilotinib use.^10^, ^11^, ^12^ Impaired glucose metabolism increases the risk of cardiovascular events and limits the patients who can receive nilotinib treatment. Furthermore, these adverse events decrease patient adherence to BCR‐ABL inhibitors. However, there have been no reports related to impaired glucose metabolism caused by imatinib or dasatinib. Imatinib, in fact, has been reported to facilitate the recovery of glucose metabolism.^13^, ^14^, ^15^ However, because these findings were reported in case reports or studies with low reliability, the effects of BCR‐ABL inhibitors on glucose metabolism remain controversial, and there is no choice but to treat this adverse event empirically. Therefore, it is important to understand this adverse event induced by BCR‐ABL inhibitors to promote their proper use. Several studies have indicated that the safety data provided by clinical trials do not reflect data from real clinical settings because of strict entry criteria or limited follow‐up durations.^9^ The long‐term follow‐up data, including the data of patients with various comorbidities in real clinical settings, are essential for understanding the accurate impact of BCR‐ABL inhibitors on glucose metabolism. However, these analyses were rarely conducted and the detailed characteristics of this adverse event remain unclear.

In recent years, spontaneous reporting systems (SRS) reflecting data from actual clinical practice have been used to evaluate drug safety. The US Food and Drug Administration (FDA) manages the FDA Adverse Event Reporting System (FAERS), which has registered more than three million spontaneous reports of adverse events and is the largest SRS database in the world.^16^ In Japan, the Pharmaceuticals and Medical Devices Agency (PMDA) manages the Japanese Adverse Drug Event Report (JADER) database, which has registered approximately 300 000 spontaneous reports of adverse events.^17^ These databases are publically available and reflect full adverse event profiles in real clinical settings. Therefore, these databases are used in pharmacovigilance analyses and are useful for evaluating the risk of adverse events reflected in real clinical settings.^18^, ^19^, ^20^, ^21^

The aim of this study was to analyze the relationship between BCR‐ABL inhibitors and impaired glucose metabolism using FAERS and the JADER database. We also evaluated the characteristics of impaired glucose metabolism induced by BCR‐ABL inhibitors.

## 2 MATERIAL AND METHODS

### 2.1 Database source

Adverse event reports were downloaded from the FDA and PMDA websites.^22^, ^23^ JADER data until May 2017, which are publicly available on the PMDA website, were used. For FAERS analysis, data from the third quarter of 2010 to the second quarter of 2015 were used. Because FAERS includes duplicate reports, only the latest report of a patient was used for analysis according to the recommendation of the FDA.^24^ Only reports with complete age and sex information were extracted. Furthermore, we analyzed reports with patient ages greater than 20 years in this analysis. Supplemental analysis using all reports was also performed. Reporting odds ratios (RORs) were used for evaluating signal detection. Each case was divided into four groups based on whether “Impaired glucose metabolism” developed and whether a BCR‐ABL inhibitor was used. Namely, there were (n11) cases who used a BCR‐ABL inhibitor and reported as “Impaired glucose metabolism,” (n12) cases who used a BCR‐ABL inhibitor and did not report as “Impaired glucose metabolism,” (n21) cases who did not use a BCR‐ABL inhibitor and reported as “Impaired glucose metabolism,” and (n22) cases who did not use a BCR‐ABL inhibitor and did not report as “Impaired glucose metabolism.” The ROR and 95% confidence interval (CI) were calculated by the following formula.^24^

$$\begin{array}{c} \text{ROR =}\;\frac{\text{n11/n21}}{\text{n12/n22}}\text{,}\;95\%\;\text{CI}=\exp\{\log\left(\text{ROR}\right)\\ \pm 1.9\text{6}\sqrt{\frac{\text{1}}{\text{n11}}\;\text{+}\;\frac{\text{1}}{\text{n12}}\;\text{+}\;\frac{\text{1}}{\text{n21}}\;\text{+}\;\frac{\text{1}}{\text{n22}}}\text{\textbackslash{}\}}\; \end{array}$$

A signal was considered detected when the lower limit of the 95% CI of the ROR exceeded one. In the subgroup analysis in JADER, reports were analyzed as 10‐year subgroups because age was described in 10‐year increments. Additionally, time‐onset analysis was performed using the drug administration start date and the adverse event occurrence date included in the JADER database. The downloaded data were processed using Microsoft Access 2016® (Microsoft, Redmond, WA). All data analyses were performed in more than two independent experiments.

### 2.2 Outcomes

In JADER and FAERS, the descriptions of adverse event names conform to the Medical Dictionary for Regulatory Activities (MedDRA) developed by the International Conference on Harmonisation of Technical Requirements for Registration of Pharmaceuticals for Human Use (ICH). Therefore, the adverse events that were defined using MedDRA conformed to the adverse event names in our study. Impaired glucose metabolism is defined by 113 preferred terms that were included in the standardized MedDRA queries “hyperglycemia/new onset diabetes mellitus” (SMQ 20000041).

### 2.3 Statistical analysis

Categorical variables are summarized in terms of frequencies and percentages. The Fisher exact test was used to compare the frequency of adverse events in the presence or absence of BCR‐ABL inhibitors. Days from the administration of nilotinib to the onset of impaired glucose metabolism in nilotinib‐administered patients were analyzed by the Kaplan‐Meier method. The analyses were performed using R statistical software version 3.3.2. Statistical significance was defined as a *P*‐value <0.05.

## 3 RESULTS

### 3.1 Reporting rate of impaired glucose metabolism reported in FAERS and the JADER database

In FAERS, 572, 306, 514, 42, and 114 cases of impaired glucose metabolism associated with imatinib, dasatinib, nilotinib, bosutinib, and ponatinib, respectively, were reported (Table 1). In the JADER database, 75, 23, 93, and three cases of impaired glucose metabolism associated with imatinib, dasatinib, nilotinib, and bosutinib, respectively, were reported. No cases associated with ponatinib were reported in the JADER database. The ROR of impaired glucose metabolism associated with nilotinib was 1.26 (95% CI: 1.149‐1.382) in FAERS and 1.32 (95% CI: 1.059‐1.634) in the JADER database, and signals for an increased reporting rate of impaired glucose metabolism were detected (*P* < 0.001). In contrast, the RORs of impaired glucose metabolism associated with imatinib and dasatinib were 0.79 (95% CI: 0.722‐0.857) and 0.80 (95% CI: 0.708‐0.896), respectively, in FAERS and 0.40 (95% CI: 0.313‐0.503) and 0.41 (95% CI: 0.261‐0.625), respectively, in the JADER database; signals for an increased reporting rate of impaired glucose metabolism were not detected. These signals were also not detected for bosutinib (ROR: 0.64, 95% CI: 0.459‐0.876) and ponatinib (ROR: 1.10, 95% CI: 0.900‐1.335) in the FAERS database.

**Table 1:** Incidences of impaired glucose metabolism induced by BCR‐ABL inhibitors reported in FAERS and the JADER database

Drug A	Impaired glucose metabolism without Drug A	Impaired glucose metabolism with Drug A	ROR	95% CI	*P* value
FAERS					
Imatinib	176 388/2 167 224 (8.86%)	572/8771 (6.52%)	0.79	0.722‐0.857	<0.001
Dasatinib	176 654/2 171 358 (8.86%)	306/4637 (6.6%)	0.80	0.708‐0.896	<0.001
Nilotinib	176 446/2 170 873 (8.85%)	514/5122 (10.04%)	1.26	1.149‐1.382	<0.001
Bosutinib	176 918/2 175 214 (8.85%)	42/781 (5.38%)	0.64	0.459‐0.876	0.004
Ponatinib	176 846/2 174 711 (8.85%)	114/1284 (8.88%)	1.1	0.9‐1.335	0.332
JADER					
Imatinib	20 064/378 563 (5.6%)	75/3429 (2.19%)	0.40	0.313‐0.503	<0.001
Dasatinib	20 116/380 973 (5.57%)	23/1019 (2.26%)	0.41	0.261‐0.625	<0.001
Nilotinib	20 046/380 634 (5.56%)	93/1358 (6.85%)	1.32	1.059‐1.634	0.012
Bosutinib	20 136/381 868 (5.57%)	3/124 (2.42%)	0.45	0.091‐1.333	0.223

CI, confidence interval; FAERS, Food and Drug Administration Adverse Event Reporting System; JADER, Japanese Adverse Drug Event Report; ROR, reporting odds ratio.

### 3.2 Relationship between impaired glucose metabolism and sex

In FAERS, signals for an increased reporting rate of impaired glucose metabolism were detected for nilotinib regardless of sex (male: ROR, 1.23, 95% CI, 1.088‐1.394; female: ROR, 1.28, 95% CI, 1.107‐1.466) (Table 2). In the JADER database, signals for an increased reporting rate of impaired glucose metabolism were detected in only the male groups treated with nilotinib (male: ROR, 1.37, 95% CI, 1.034‐1.783; female: ROR, 1.23, 95% CI, 0.839‐1.757). These signals were not detected for other BCR‐ABL inhibitors regardless of sex.

**Table 2:** Effect of sex on impaired glucose metabolism reported in FAERS and the JADER database

Drug A	Impaired glucose metabolism without Drug A	Impaired glucose metabolism with Drug A	ROR	95% CI	*P* value
FAERS					
Male					
Imatinib	67 905/815 539 (9.08%)	266/4850 (5.48%)	0.64	0.562‐0.723	<0.001
Dasatinib	68 023/818 027 (9.07%)	148/2362 (6.27%)	0.74	0.62‐0.871	<0.001
Nilotinib	67 882/817 513 (9.06%)	289/2876 (10.05%)	1.23	1.088‐1.394	0.001
Bosutinib	68 151/820 016 (9.06%)	20/373 (5.36%)	0.63	0.377‐0.98	0.039
Ponatinib	68 115/819 727 (9.06%)	56/662 (8.46%)	1.02	0.761‐1.342	0.888
Female					
Imatinib	108 483/1 351 685 (8.73%)	306/3921 (7.8%)	0.97	0.86‐1.091	0.638
Dasatinib	108 631/1 353 331 (8.73%)	158/2275 (6.95%)	0.86	0.723‐1.006	0.058
Nilotinib	108 564/1 353 360 (8.72%)	225/2246 (10.02%)	1.28	1.107‐1.466	<0.001
Bosutinib	108 767/1 355 198 (8.73%)	22/408 (5.39%)	0.65	0.405‐1.003	0.055
Ponatinib	108 731/1 354 984 (8.72%)	58/622 (9.32%)	1.18	0.883‐1.546	0.237
JADER					
Male					
Imatinib	10 395/192 651 (5.7%)	35/1926 (1.82%)	0.32	0.225‐0.453	<0.001
Dasatinib	10 418/194 001 (5.67%)	12/576 (2.08%)	0.37	0.193‐0.66	<0.001
Nilotinib	10 370/193 742 (5.66%)	60/835 (7.19%)	1.37	1.034‐1.783	0.025
Bosutinib	10 427/194 499 (5.66%)	3/78 (3.85%)	0.71	0.142‐2.145	0.800
Female					
Imatinib	9669/185 912 (5.49%)	40/1503 (2.66%)	0.5	0.354‐0.682	<0.001
Dasatinib	9698/186 972 (5.47%)	11/443 (2.48%)	0.47	0.231‐0.842	0.007
Nilotinib	9676/186 892 (5.46%)	33/523 (6.31%)	1.23	0.839‐1.757	0.235
Bosutinib	7162/157 401 (4.77%)	0/41 (0%)	‐	‐	‐

CI, confidence interval; FAERS, Food and Drug Administration Adverse Event Reporting System; JADER, Japanese Adverse Drug Event Report; ROR, reporting odds ratio.

### 3.3 Relationship between impaired glucose metabolism and age

In FAERS, signals for an increased reporting rate of impaired glucose metabolism were detected for nilotinib regardless of age (20‐60 s: ROR, 1.16, 95% CI, 1.031‐1.299; 70‐90 s: ROR, 1.47, 95% CI, 1.257‐1.714) (Table 3). In the JADER database, signals for an increased reporting rate of impaired glucose metabolism were detected in only the younger group treated with nilotinib (20‐60 s: ROR, 1.51, 95% CI, 1.127‐1.991; 70‐90 s: ROR, 1.11, 95% CI, 0.779‐1.550). These signals were not detected for other BCR‐ABL inhibitors regardless of age.

**Table 3:** Effect of age on impaired glucose metabolism reported in FAERS and the JADER database

Drug A	Impaired glucose metabolism without Drug A	Impaired glucose metabolism with Drug A	ROR	95% CI	*P* value
FAERS					
20‐60 s					
Imatinib	132 131/1 641 998 (8.75%)	393/6392 (6.15%)	0.75	0.674‐0.829	<0.001
Dasatinib	132 304/1 645 007 (8.75%)	220/3383 (6.5%)	0.8	0.69‐0.912	<0.001
Nilotinib	132 199/1 644 856 (8.74%)	325/3534 (9.2%)	1.16	1.031‐1.299	0.013
Bosutinib	132 497/1 647 848 (8.74%)	27/542 (4.98%)	0.6	0.391‐0.883	0.007
Ponatinib	132 444/1 647 417 (8.74%)	80/973 (8.22%)	1.02	0.805‐1.289	0.814
70‐90 s					
Imatinib	44 257/525 227 (9.2%)	179/2379 (7.52%)	0.88	0.755‐1.031	0.120
Dasatinib	44 350/526 352 (9.2%)	86/1254 (6.86%)	0.8	0.635‐0.997	0.047
Nilotinib	44 247/526 018 (9.18%)	189/1588 (11.9%)	1.47	1.257‐1.714	<0.001
Bosutinib	44 421/527 367 (9.2%)	15/239 (6.28%)	0.73	0.401‐1.227	0.293
Ponatinib	44 402/527 295 (9.19%)	34/311 (10.93%)	1.33	0.906‐1.911	0.124
JADER					
20‐60 s					
Imatinib	10 542/223 081 (4.96%)	40/2088 (1.92%)	0.39	0.28‐0.538	<0.001
Dasatinib	10 565/224 491 (4.94%)	17/678 (2.51%)	0.52	0.301‐0.841	0.005
Nilotinib	10 527/224 375 (4.92%)	55/794 (6.93%)	1.51	1.127‐1.991	0.005
Bosutinib	10 581/225 096 (4.93%)	1/73 (1.37%)	0.28	0.007‐1.62	0.265
70‐90 s					
Imatinib	9522/155 482 (6.52%)	35/1341 (2.61%)	0.41	0.285‐0.575	<0.001
Dasatinib	9551/156 482 (6.5%)	6/341 (1.76%)	0.28	0.1‐0.606	<0.001
Nilotinib	9519/156 259 (6.49%)	38/564 (6.74%)	1.11	0.779‐1.55	0.536
Bosutinib	9555/156 772 (6.49%)	2/51 (3.92%)	0.63	0.074‐2.397	0.770

CI, confidence interval; FAERS, Food and Drug Administration Adverse Event Reporting System; JADER, Japanese Adverse Drug Event Report; ROR, reporting odds ratio.

### 3.4 Time‐onset analysis of impaired glucose metabolism using the JADER database

**Figure 1:**
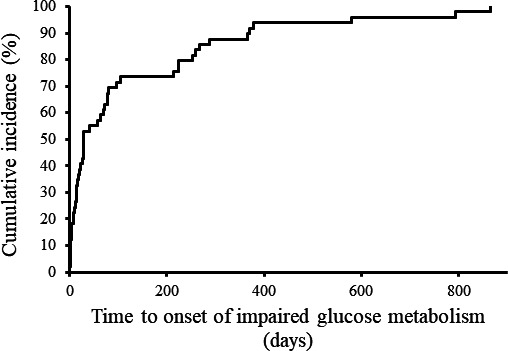
Time‐onset analysis of impaired glucose metabolism by using the JADER database. Kaplan‐Meier plot from the start of drug administration to the time of reporting of impaired glucose metabolism

The time‐onset analysis showed that the reports of impaired glucose metabolism induced by nilotinib increased within 200 days. However, late‐onset events beyond 600 days were also reported (Figure 1).

## 4 DISCUSSION

Our analyses using FAERS and the JADER database revealed that signals for an increased reporting rate were detected only for nilotinib. These signals were clearly detected in male or younger patients. However, these signals were not detected for other BCR‐ABL inhibitors.

Our study showed the relationship between nilotinib and the increased reporting rate of impaired glucose metabolism. However, the mechanism of impaired glucose metabolism induced by nilotinib remains unclear. Some case reports have suggested that nilotinib decreases insulin sensitivity or inhibits insulin secretion, causing impaired glucose metabolism.^25^, ^26^ However, because only a few studies have referred to this phenomenon, the increase in the risk of impaired glucose metabolism induced by nilotinib was evaluated empirically herein. Signals of increased reporting rate were detected for nilotinib in the analysis comprising all patient reports in FAERS, and this trend was also observed in the analysis based on the JADER database (Table S1). Therefore, this result is considered valid. Follow‐up analysis for 5 years in the patients who received nilotinib or imatinib revealed that increased glucose levels were clearly observed in nilotinib‐treated patients, which is consistent with our results.^27^ To our knowledge, this study is the first to demonstrate the relationship between nilotinib and increased risk of impaired glucose metabolism. A reduction in the risk of cardiovascular events is necessary to maintain the quality of life during CML treatment.^28^ Our study suggests avoiding nilotinib treatment for patients with diabetes or other diseases that increase the risk of cardiovascular events. The time‐onset analysis revealed that impaired glucose metabolism induced by nilotinib was reported at more than a year. This result highlights the importance of continuous monitoring of blood glucose levels during nilotinib treatment. The subgroup analyses revealed that the signals of increased reporting rate of impaired glucose metabolism induced by nilotinib were clearly detected in male or younger patients. BCR‐ABL inhibitor treatment lasts a lifetime. Therefore, it may be better to avoid nilotinib treatment in younger patients with a high risk of diabetes mellitus.

Decreased adherence to BCR‐ABL inhibitor treatment commonly occurs.^29^ Although age and sex were reported as the factors for limiting adherence, adverse events induced by BCR‐ABL inhibitors also decrease adherence.^30^ Early detection of adverse events and adequate supportive care is useful to improve adherence. Moreover, Leader et al^31^ reported that multidisciplinary intervention improved the adherence to BCR‐ABL inhibitors. Our studies provide useful information for conducting optimal supportive care to improve this adherence.

There are some limitations associated with FAERS and the JADER database because these databases are reporting systems for spontaneous adverse events. Data from these databases were collected from various clinical settings wherein adverse event reporting was voluntary. Therefore, these databases might include duplicate data.^32^ We excluded duplicate data according to the recommendation of the FDA. In addition to the possibility of an over‐reporting bias, an under‐reporting bias, due to nonreporting of adverse events by health care workers, exists.^32^ It is known that spontaneous adverse event reports tend to increase after drug safety alerts or publications, which is called notoriety bias.^33^ Considering these reporting biases, caution should be exercised when interpreting results obtained from only one SRS database. In our study, we used two SRS databases, FAERS and the JADER database, to overcome reporting biases and increase the reliability of our results. FAERS is one of the largest SRS databases in the world and is suitable for analyzing the risk detection of adverse events induced by medications, owing to the large number of reports. However, this database does not include detailed data pertaining to adverse events, such as durations of medication administration and patient characteristics. In contrast, the JADER database contains detailed patient characteristics and adverse events, although the number of reports is lower than that in FAERS because the JADER database contains data from only Japanese patients. Our results have high reliability because the same results were obtained from two databases. Although we recognize the limitations associated with SRS, our study provides useful evidence showing differences in the effects on impaired glucose metabolism between BCR‐ABL inhibitors.

We used RORs to detect signals of risk increase or decrease. In data mining analyses, proportional reporting ratios (PRR) and multi‐item gamma Poisson Shrinker (MGPS) are used for signal detection algorithms.^9^, ^32^ Although all algorithms showed high specificity, each algorithm showed different sensitivity.^34^ The detection sensitivity of MGPS is 26%, and we should consider overlooking risk when using MGPS‐based algorithms. On the contrary, ROR and PRR have high sensitivity. Especially, ROR is usually used in signal detection algorithms due to its simplicity.^17^, ^21^, ^24^ However, ROR‐based algorithms have the disadvantage of decreased detection power when the number of adverse event reports is small. The number of reports of impaired glucose metabolism related to BCR‐ABL inhibitors was not small, and it was reasonable to use ROR in signal detection in our study. A high ROR indicates an increase in the risk of an adverse event report, not an increase in the risk of development of adverse events. Therefore, we are aware that an increased ROR only offers a rough indication of signal strength.

Despite the inherent limitations of signal detection using SRS databases, our results regarding the differences in the effects on impaired glucose metabolism between BCR‐ABL inhibitors are in agreement with the results of previous studies. Thus, our study provides beneficial information for the management of adverse events induced by BCR‐ABL inhibitors.

## 5 CONCLUSION

Our study using FAERS and the JADER database revealed that the use of nilotinib increased the reporting rate of impaired glucose metabolism, whereas other BCR‐ABL inhibitors did not increase reports of this adverse event. This finding indicated that only nilotinib increased the risk of impaired glucose tolerance and that adverse events might be drug effects of nilotinib and not class effects of BCR‐ABL inhibitors. Although further analyses are required to confirm these findings, our results indicated differences in the effect on glucose metabolism between BCR‐ABL inhibitors and that careful monitoring of glucose levels is necessary among nilotinib‐treated patients.

## CONFLICT OF INTEREST

The authors have indicated that they have no conflicts of interest regarding the content of this article.

## References

[1] Druker BJ , Sawyers CL , Kantarjian H , et al. Activity of a specific inhibitor of the BCR‐ABL tyrosine kinase in the blast crisis of chronic myeloid leukemia and acute lymphoblastic leukemia with the Philadelphia chromosome. N Engl J Med. 2001;344(14):1038‐1042. 11287973 10.1056/NEJM200104053441402

[2] Druker BJ , Guilhot F , O'Brien SG , et al. Five‐year follow‐up of patients receiving imatinib for chronic myeloid leukemia. N Engl J Med. 2006;355(23):2408‐2417. 17151364 10.1056/NEJMoa062867

[3] Kantarjian HM , Hochhaus A , Saglio G , et al. Nilotinib versus imatinib for the treatment of patients with newly diagnosed chronic phase, Philadelphia chromosome‐positive, chronic myeloid leukaemia: 24‐month minimum follow‐up of the phase 3 randomised ENESTnd trial. Lancet Oncol. 2011;12(9):841‐851. 21856226 10.1016/S1470-2045(11)70201-7

[4] Kantarjian H , Shah NP , Hochhaus A , et al. Dasatinib versus imatinib in newly diagnosed chronic‐phase chronic myeloid leukemia. N Engl J Med. 2010;362(24):2260‐2270. 20525995 10.1056/NEJMoa1002315

[5] Cortes JE , Kantarjian H , Shah NP , et al. Ponatinib in refractory Philadelphia chromosome‐positive leukemias. N Engl J Med. 2012;367(22):2075‐2088. 23190221 10.1056/NEJMoa1205127 PMC3777383

[6] Cortes JE , Kantarjian HM , Brummendorf TH , et al. Safety and efficacy of bosutinib (SKI‐606) in chronic phase Philadelphia chromosome‐positive chronic myeloid leukemia patients with resistance or intolerance to imatinib. Blood. 2011;118(17):4567‐4576. 21865346 10.1182/blood-2011-05-355594 PMC4916618

[7] Noens L , van Lierde MA , De Bock R , et al. Prevalence, determinants, and outcomes of nonadherence to imatinib therapy in patients with chronic myeloid leukemia: the ADAGIO study. Blood. 2009;113(22):5401‐5411. 19349618 10.1182/blood-2008-12-196543

[8] Demetri GD , Wang Y , Wehrle E , et al. Imatinib plasma levels are correlated with clinical benefit in patients with unresectable/metastatic gastrointestinal stromal tumors. J Clin Oncol. 2009;27(19):3141‐3147. 19451435 10.1200/JCO.2008.20.4818

[9] Cortes J , Mauro M , Steegmann JL , et al. Cardiovascular and pulmonary adverse events in patients treated with BCR‐ABL inhibitors: Data from the FDA Adverse Event Reporting System. Am J Hematol. 2015;90(4):E66–E72. 25580915 10.1002/ajh.23938 PMC11458256

[10] Racil Z , Razga F , Drapalova J , et al. Mechanism of impaired glucose metabolism during nilotinib therapy in patients with chronic myelogenous leukemia. Haematologica. 2013;98(10):e124‐e126. 23716549 10.3324/haematol.2013.086355 PMC3789473

[11] Iurlo A , Orsi E , Cattaneo D , et al. Effects of first‐ and second‐generation tyrosine kinase inhibitor therapy on glucose and lipid metabolism in chronic myeloid leukemia patients: a real clinical problem? Oncotarget. 2015;6(32):33944‐33951. 26376678 10.18632/oncotarget.5580 PMC4741815

[12] Breccia M , Muscaritoli M , Gentilini F , et al. Impaired fasting glucose level as metabolic side effect of nilotinib in non‐diabetic chronic myeloid leukemia patients resistant to imatinib. Leuk Res. 2007;31(12):1770‐1772. 17382388 10.1016/j.leukres.2007.01.024

[13] Ono K , Suzushima H , Watanabe Y , et al. Rapid amelioration of hyperglycemia facilitated by dasatinib in a chronic myeloid leukemia patient with type 2 diabetes mellitus. Int Med. 2012;51(19):2763‐2766. 10.2169/internalmedicine.51.8314 23037470

[14] Breccia M , Muscaritoli M , Cannella L , Stefanizzi C , Frustaci A , Alimena G . Fasting glucose improvement under dasatinib treatment in an accelerated phase chronic myeloid leukemia patient unresponsive to imatinib and nilotinib. Leuk Res. 2008;32(10):1626‐1628. 18321570 10.1016/j.leukres.2008.01.015

[15] Agostino NM , Chinchilli VM , Lynch CJ , et al. Effect of the tyrosine kinase inhibitors (sunitinib, sorafenib, dasatinib, and imatinib) on blood glucose levels in diabetic and nondiabetic patients in general clinical practice. J Oncol Pharm Pract. 2011;17(3):197‐202. 20685771 10.1177/1078155210378913

[16] Harpaz R , DuMouchel W , LePendu P , Bauer‐Mehren A , Ryan P , Shah NH . Performance of pharmacovigilance signal‐detection algorithms for the FDA adverse event reporting system. Clin Pharmacol Ther. 2013;93(6):539‐546. 23571771 10.1038/clpt.2013.24 PMC3857139

[17] Takeyama M , Sai K , Imatoh T , Segawa K , Hirasawa N , Saito Y . Influence of Japanese regulatory action on denosumab‐related hypocalcemia using japanese adverse drug event report database. Biol Pharm Bull. 2017;40(9):1447‐1453. 28867727 10.1248/bpb.b17-00266

[18] Welch HK , Kellum JA , Kane‐Gill SL . Drug‐associated acute kidney injury identified in the united states food and drug administration adverse event reporting system database. Pharmacotherapy. 2018;38(8):785‐793. 29883524 10.1002/phar.2152

[19] Sanagawa A , Hotta Y , Kataoka T , et al. Hepatitis B infection reported with cancer chemotherapy: analyzing the US FDA adverse event reporting system. Cancer Med. 2018;7(6):2269‐2279. 29663729 10.1002/cam4.1429 PMC6010750

[20] Noguchi Y , Katsuno H , Ueno A , et al. Signals of gastroesophageal reflux disease caused by incretin‐based drugs: a disproportionality analysis using the Japanese adverse drug event report database. J Pharm Health Care Sci. 2018;4:15. 29946474 10.1186/s40780-018-0109-z PMC6004661

[21] Fukuda A , Tahara K , Hane Y , et al. Comparison of the adverse event profiles of conventional and liposomal formulations of doxorubicin using the FDA adverse event reporting system. PLoS ONE. 2017;12(9):e0185654. 28953936 10.1371/journal.pone.0185654 PMC5617225

[22] US Food and Drug Administration . FDA Adverse Event Reporting System web site. https://www.fda.gov/Drugs/GuidanceComplianceRegulatoryInformation/Surveillance/AdverseDrugEffects/ucm082193.htm. Accessed June 5, 2018.

[23] Pharmaceuticals and Medical Devices Agency . Japanese Adverse Drug Event Report database web site. https://www.pmda.go.jp/safety/info-services/drugs/adr-info/suspected-adr/0003.html. Accessed June 5, 2018.

[24] Suzuki Y , Suzuki H , Umetsu R , et al. Analysis of the interaction between clopidogrel, aspirin, and proton pump inhibitors using the FDA adverse event reporting system database. Biol Pharm Bull. 2015;38(5):680‐686. 25947914 10.1248/bpb.b14-00191

[25] Ito Y , Miyamoto T , Chong Y , Maki T , Akashi K , Kamimura T . Nilotinib exacerbates diabetes mellitus by decreasing secretion of endogenous insulin. Int J Hematol. 2013;97(1):135‐138. 23179903 10.1007/s12185-012-1222-7

[26] Breccia M , Loglisci G , Salaroli A , Serrao A , Alimena G . Nilotinib‐mediated increase in fasting glucose level is reversible, does not convert to type 2 diabetes and is likely correlated with increased body mass index. Leuk Res. 2012;36(4):e66‐e67. 22218012 10.1016/j.leukres.2011.12.011

[27] Hochhaus A , Saglio G , Hughes TP , et al. Long‐term benefits and risks of frontline nilotinib vs imatinib for chronic myeloid leukemia in chronic phase: 5‐year update of the randomized ENESTnd trial. Leukemia. 2016;30(5):1044‐1054. 26837842 10.1038/leu.2016.5 PMC4858585

[28] Douxfils J , Haguet H , Mullier F , Chatelain C , Graux C , Dogne JM . Association between BCR‐ABL tyrosine kinase inhibitors for chronic myeloid leukemia and cardiovascular events, major molecular response, and overall survival: a systematic review and meta‐analysis. JAMA Oncol. 2016;2(5):625‐632. 10.1001/jamaoncol.2015.5932 26847662

[29] Rychter A , Jerzmanowski P , Holub A , et al. Treatment adherence in chronic myeloid leukaemia patients receiving tyrosine kinase inhibitors. Med Oncol. 2017;34(6):104. 28444623 10.1007/s12032-017-0958-6 PMC5405100

[30] Jabbour EJ , Kantarjian H , Eliasson L , Cornelison AM , Marin D . Patient adherence to tyrosine kinase inhibitor therapy in chronic myeloid leukemia. Am J Hematol. 2012;87(7):687‐691. 22473898 10.1002/ajh.23180 PMC11726351

[31] Leader A , Benyamini N , Gafter‐Gvili A , et al. Effect of adherence‐enhancing interventions on adherence to tyrosine kinase inhibitor treatment in chronic myeloid leukemia (TAKE‐IT): a quasi‐experimental pre‐post intervention multicenter pilot study. Clin Lymphoma Myeloma Leuk. 2018;18(11):e449‐e461. 30030034 10.1016/j.clml.2018.06.026

[32] Bate A , Evans SJ . Quantitative signal detection using spontaneous ADR reporting. Pharmacoepidemiol Drug Saf. 2009;18(6):427‐436. 19358225 10.1002/pds.1742

[33] de Boissieu P , Kanagaratnam L , Abou Taam M , Roux MP , Drame M , Trenque T . Notoriety bias in a database of spontaneous reports: the example of osteonecrosis of the jaw under bisphosphonate therapy in the French national pharmacovigilance database. Pharmacoepidemiol Drug Saf. 2014;23(9):989‐992. 24737486 10.1002/pds.3622

[34] Matsushita Y , Kuroda Y , Niwa S , Sonehara S , Hamada C , Yoshimura I . Criteria revision and performance comparison of three methods of signal detection applied to the spontaneous reporting database of a pharmaceutical manufacturer. Drug Saf. 2007;30(8):715‐726. 17696584 10.2165/00002018-200730080-00008

