# Supplementary material for: Pharmacovigilance evaluation of the relationship between impaired glucose metabolism and BCR‐ABL inhibitor use by using an adverse drug event reporting database
Source: Cancer Med. 2018 Dec 18;8(1):174–81. doi: 10.1002/cam4.1920 (PMC6346261; doi:10.1002/cam4.1920)
Supplement: Supplementary file 1 [file CAM4-8-174-s001.docx]

**Supplemental Table 1. Supplemental analysis of incidences of impaired glucose metabolism induced by BCR-ABL inhibitors using all reports in FAERS and the JADER database**

| **Drug A** | **Impaired glucose metabolism without Drug A** | **Impaired glucose metabolism with Drug A** | **ROR** | **95% CI** | **p value** |
| --- | --- | --- | --- | --- | --- |
| FAERS |  |  |  |  |  |
| Imatinib | 267704/3705541 (7.8%) | 897/18213 (4.9%) | 0.67 | 0.621–0.712 | <0.001 |
| Dasatinib | 268197/3716583 (7.8%) | 404/7171 (5.6%) | 0.77 | 0.693–0.849 | <0.001 |
| Nilotinib | 267874/3714991 (7.8%) | 727/8763 (8.3%) | 1.16 | 1.078–1.256 | <0.001 |
| Bosutinib | 268550/3722755 (7.8%) | 51/999 (5.1%) | 0.69 | 0.511–0.917 | 0.008 |
| Ponatinib | 268453/3721956 (7.8%) | 148/1798 (8.2%) | 1.15 | 0.969–1.366 | 0.100 |
| JADER |  |  |  |  |  |
| Imatinib | 23672/445264 (5.61%) | 86/4294 (2%) | 0.36 | 0.29–0.451 | <0.001 |
| Dasatinib | 23734/448449 (5.59%) | 24/1109 (2.16%) | 0.40 | 0.252–0.592 | <0.001 |
| Nilotinib | 23652/447855 (5.58%) | 106/1703 (6.22%) | 1.19 | 0.968–1.45 | 0.092 |
| Bosutinib | 23755/449405 (5.58%) | 3/153 (1.96%) | 0.36 | 0.073–1.068 | 0.069 |

Abbreviations: JADER: Japanese Adverse Drug Event Report, FAERS: Food and Drug Administration Adverse Event Reporting System, ROR: reporting odds ratio, CI: confidence interval
